# Acute Lung Injury As Severe Acute Respiratory Distress Syndrome After Fentanyl Overdose

**DOI:** 10.7759/cureus.52745

**Published:** 2024-01-22

**Authors:** Asha Bansari, Han Li, Sai Chunduru, Naveen Baskaran

**Affiliations:** 1 Department of Medicine, University of Florida College of Medicine, Gainesville, USA; 2 Department of Dermatology, University of Florida College of Medicine, Gainesville, USA; 3 Department of Medicine, Osmania Medical College, Hyderabad, IND

**Keywords:** acute respiratory distress syndrome (ards), opioid side effects, lung-injury, drug intoxication, drug overdose, illicit fentanyl

## Abstract

Acute lung injury following fentanyl overdose is an unusual presentation. Pulmonary edema has been associated with opioid and naloxone use. However, to our knowledge, there have been no previous reports of inhaled fentanyl-associated acute lung injury presenting with acute hypoxic respiratory failure secondary to severe acute respiratory distress syndrome. We report a case of inhaled fentanyl-related severe acute respiratory distress syndrome which presented immediately after snorting fentanyl. This patient developed hypoxia requiring 100% oxygen on non-rebreather mask, and acute respiratory distress syndrome was confirmed on chest X-ray and computed tomography on admission. He was successfully treated with steroids with recovery in 48 hours. Naloxone was used in this patient, which has been associated with pulmonary edema in case reports and series, but clinical findings were more consistent with acute respiratory distress syndrome rather than pulmonary edema. The mechanism for this clinical presentation is not well known. Proposed mechanisms include lung injury from inhalation against an obstruction in a manner similar to post-obstructive pulmonary edema. Although our patient rapidly responded to symptomatic treatment and steroid course, our case also highlights the need for further study to elucidate the various clinical presentations associated with fentanyl use-related lung toxicity including acute respiratory distress syndrome.

## Introduction

Fentanyl is a potent synthetic opioid most commonly associated with overdose-related mortality. Lately, illicit use of opioids, particularly fentanyl, is on the rise as an epidemic. We report a case of a man in his 20s presenting with acute respiratory distress after inhaled fentanyl use. Although our patient had received naloxone, the imaging and clinical course essentially ruled out naloxone-induced pulmonary edema. Given the increasing frequency of opioid abuse, physicians should be aware of the clinical presentations related to the potential toxic effects of opioids as well as its reversing agent naloxone. More specifically targeted studies are needed to explore the pathophysiology and causal relationship between fentanyl and acute lung injury.

## Case presentation

**Figure 1:**
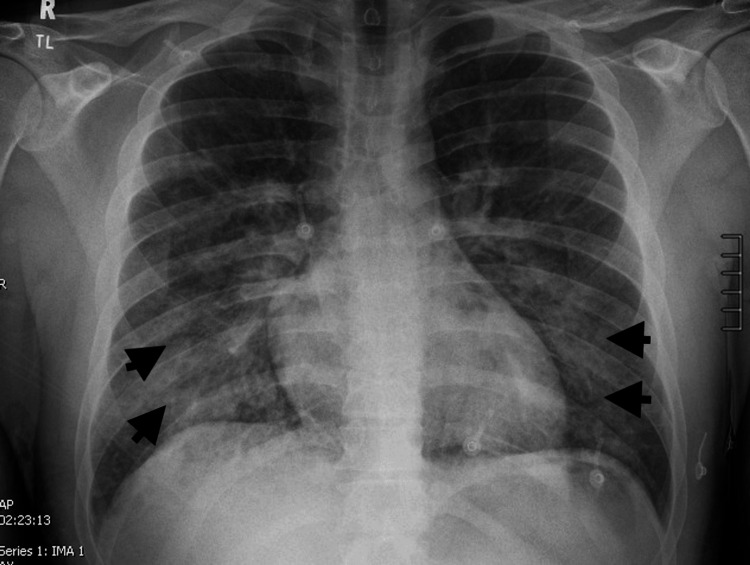
Bilateral mid to lower lung opacities on presentation denoted by black arrows

**Figure 2:**
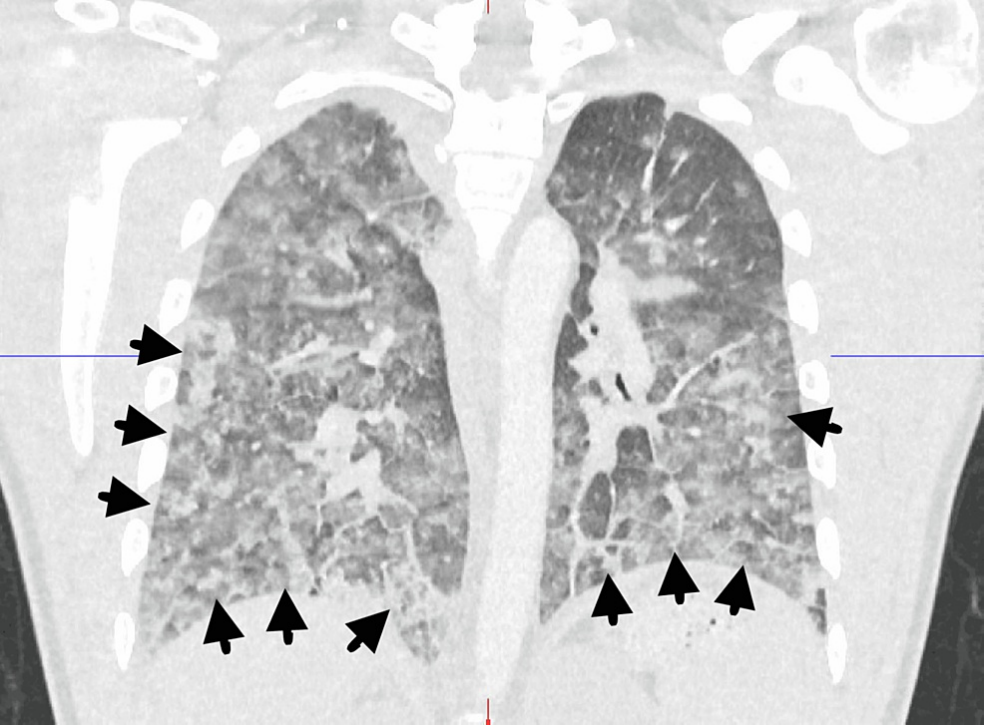
CT chest with contrast on initial presentation showing patchy consolidations and ground glass opacities in both lungs with basilar predominance denoted by black arrows

**Figure 3:**
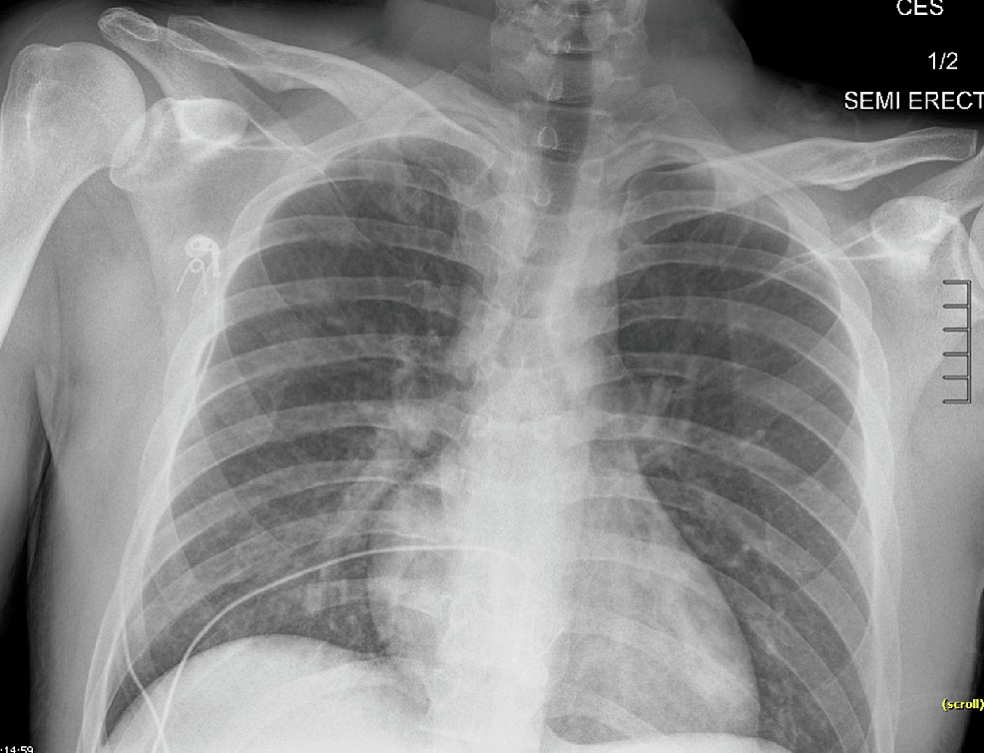
Resolving bilateral airspace disease after 24 hours

A young male in his 20s with no significant past medical history presented from prison for respiratory distress after snorting fentanyl. In the prison medical unit, he was given two doses of 0.4 mg intranasal naloxone with improvement in mentation. While there, he had shortness of breath requiring 100% oxygen via a non-rebreather mask, and was transferred to the emergency room. In addition to respiratory distress, he acquired a productive cough without hemoptysis. He denied fever, chills, chest pain, dizziness, and suicidal ideation. On admission, he had bilateral diminished breath sounds with no crackles or rhonchi and a small superficial laceration to the right brow. Initial vitals were significant for a BP of 92/65 mmHg and oxygen saturation of 89% on the non-rebreather mask at 15 L/minute. The COVID-19 test was negative. PaO_2_/FiO_2_ ratio was 57.14. The urine drug screen was positive for fentanyl without any other drugs. Chest X-ray showed bilateral mid-to-lower lung airspace opacities (Figure 1), consistent with acute lung injury and pulmonary edema. Computed tomography (CT) of the chest with contrast on initial presentation showed patchy consolidations and ground glass opacities with bilateral basilar predominance (Figure 2), consistent with noncardiogenic pulmonary edema or acute lung injury. The patient was treated with a high-flow nasal cannula at a FiO_2_ of 55% and flow rate of 45 L/minute with improvement in his saturation to 95%-100% and dexamethasone 6 mg daily intravenously for five days. The patient significantly improved with supportive care, and a subsequent chest X-ray showed resolution of airspace disease after 24 hours of treatment (Figure 3). He maintained saturations >94% on room air on hospital day three. The patient was discharged on hospital day three with clinical and radiological improvement and no need for supplemental oxygen.

## Discussion

Fentanyl overdose is a rising health crisis. Prescription fentanyl is available in pill, lozenge, nasal or sublingual spray, and non-pharmaceutical fentanyl is manufactured illegally in a powder, liquid, or tablet form often mixed with other illicit drugs like cocaine, methamphetamine, and heroin [1]. Our patient used the white powder form, which was confirmed to be fentanyl on chemical testing with no additional drugs or impurities. Fentanyl overdose may present as small constricted bilateral pinpoint pupils, choking, cold and/or clammy skin, loss of consciousness with respiratory depression with slow, weak, or absent breathing. Interestingly, our patient’s chest CT scan was concerning for acute lung injury which has been reported to be associated with cocaine [2,3]. There were no reported cases of such clinical presentation with inhaled fentanyl use. However, there were few case reports of fentanyl-induced diffuse alveolar hemorrhage [4,5]. There have been reports of diffuse alveolar hemorrhage following opioid overdose on autopsies [6,7]. Diffuse alveolar hemorrhage remains a possible underlying etiology in our patient, as 1) acute lung injury may lead to diffuse alveolar hemorrhage, 2) diffuse alveolar hemorrhage has nonspecific radiological findings that resemble acute lung injury, 3) and bronchoscopy was not performed to distinguish the two. However, our patient’s lack of hemoptysis and normal hemoglobin and white counts make acute lung injury more likely.

Given our patient had received naloxone prior to arrival at the emergency room, we reviewed the literature regarding naloxone-associated lung injury as well. A case series of 10 patients reported naloxone-associated pulmonary edema following opioid overdose [8]. The proposed mechanism for naloxone-associated pulmonary edema was described as sympathetic overdrive with opioid reversal leading to abrupt increase in pulmonary blood flow and increased permeability of capillaries [8]. Another case series reported dose-dependence to the risk of naloxone-induced pulmonary edema, which was managed with diuresis [9,10]. Our patient did not have clinical findings concerning pulmonary edema; on the contrary, our patient improved with steroids, which usually worsens volume overload states. In contrast, the pathophysiology of opioid-induced lung injury is unknown; proposed mechanisms include a combination of direct toxicity, histamine release, hypoxia, and acidosis causing increased permeability of pulmonary vasculature [11]. Another mechanism called post-obstructive pulmonary edema (POPE) has been reported to be associated with opioid use. It has been described in two forms: POPE I occurs secondary to upper airway obstruction from various causes, including strangulation, near-drowning, choking, or potentially as an indirect result of opioid overdose leading to lung injury in the form of pulmonary edema. The pathogenesis of POPE I is complex and involves multiple factors. These can include inhaling against an obstruction, which creates negative intrathoracic pressure, increasing venous return, and reducing cardiac output. This can lead to the transudation of fluid into the lungs. Additional contributing factors may include premature extubation, a short neck, obesity, narcotic use, vocal cord paralysis, and endotracheal tube obstruction [12].

POPE II occurs after surgical relief of an upper airway obstruction. The underlying pathophysiology is less well understood, but it is thought to involve an obstructive lesion that generates a modest positive end-expiratory pressure (PEEP). Once this obstruction is relieved, altered permeability can result in the transudation of interstitial fluid, leading to pulmonary edema [12,13]. A case-control study of six ICU patients showed acute respiratory distress syndrome associated with epidural fentanyl infusion [14].

## Conclusions

We described the rare case of acute respiratory distress syndrome from inhaled fentanyl use. Given the rising epidemic of fentanyl abuse, this report is of particular importance to alert clinicians of this rare yet serious adverse effect of inhaled fentanyl use. Other opioids, as well as naloxone, have been reported in association with pulmonary complications and should be considered in evaluating the patient with opioid overdose. Future studies are necessary to elucidate the underlying pathophysiology, which could lead to targeted therapeutics or preventive agents.
